# Dysfunctional personality traits and body image in orthopedic surgery patients at risk of postoperative psychological distress

**DOI:** 10.1038/s41598-025-28409-4

**Published:** 2025-12-24

**Authors:** Katarzyna Brzezewska, Wiktoria Walenista, Jolanta Starosta, Sebastian Lizińczyk, Agnieszka Bandura, Zofia Bernasik-Smagała, Julian Dutka, Bernadetta Izydorczyk

**Affiliations:** 1 Doctoral School of Social Sciences, Jagiellonian University, Kraków, Poland; 2 Institute of Psychology, Faculty of Philosophy, Jagiellonian University, Kraków, Poland; 3 Institute of Applied Psychology, Faculty of Management and Social Communication, Jagiellonian University, Kraków, Poland; 4 Department of Orthopedic and Trauma Surgery, Stefan Żeromski Specialist Hospital in Kraków, Kraków, Poland

**Keywords:** Psychology, Health care, Public health, Quality of life

## Abstract

Orthopedic surgery disrupts body integrity and often results in pain, reduced mobility, and temporary loss of autonomy, potentially triggering psychological distress and altered body image. Despite growing interest in postoperative psychological outcomes, the role of dysfunctional personality traits in shaping body image remains underexplored. This exploratory, cross-sectional observational study aimed to examine whether specific dysfunctional personality traits are associated with distinct aspects of body image in 53 adult orthopedic surgery patients assessed within 72 h post-surgery in a Polish hospital. The Personality Inventory for ICD-11 and the Multidimensional Body-Self Relations Questionnaire were used. Progressive stepwise regression analysis showed that dissociality was positively associated with appearance evaluation (β = 0.38, *p* < 0.01, R² = 0.13) and fitness evaluation (β = 0.36, *p* < 0.01, R² = 0.11). Negative affectivity was negatively associated with health evaluation (β = − 0.35, *p* < 0.05, R² = 0.08) and positively associated with weight evaluation (β = 0.40, *p* < 0.05, R² = 0.09). Anankastia was positively related to health orientation (β = 0.29, *p* < 0.05, R² = 0.07) and illness orientation (β = 0.31, *p* < 0.05, R² = 0.06). These findings suggest that dysfunctional personality traits are differentially related to body image dimensions shortly after surgery. Early personality screening may help identify patients at risk for body image disturbances and support the implementation of targeted psychological interventions, such as psychoeducational programs, to enhance emotional adjustment and, if confirmed in future research, optimize recovery outcomes.

**Supplementary Information:**

The online version contains supplementary material available at 10.1038/s41598-025-28409-4.

## Introduction

### Body image: clinical significance and psychological predictors

Body image is a multidimensional construct that encompasses individuals’ thoughts, beliefs, feelings, and behaviours related to their body and physical functioning^1,2^. Negative body image has been associated with a wide range of psychological problems, including depression^3^, eating disorders^4^, sexual dysfunctions^5^, disturbed identity formation^6^, and overall psychological distress^7^. These associations have been confirmed in both clinical^8^ and non-clinical^9^ populations. The relevance of body image in psychological functioning is further underscored by the consistently high levels of body dissatisfaction reported across diverse populations, including adolescent girls and adult men^10,11^.

Body image is shaped by various psychological and social factors. Sociocultural appearance ideals^9^, parenting style and family dynamics^12^, peer influence^13^, and biological sex^14^ all contribute to its development. Emotional regulation and coping strategies are also significant influences. Maladaptive emotion regulation strategies, including emotional eating and excessive exercise, have been associated with greater body dissatisfaction and problematic health behaviours^15,16^.

### Personality traits and body image

An increasing body of research has examined how personality traits relate to body image. Studies based on the Five-Factor Model (FFM) have consistently demonstrated associations between neuroticism, extraversion, and body image^17–19^. Neuroticism appears to have the strongest association with negative body evaluations; individuals high in this trait tend to report more negative body evaluations^17^. Neuroticism is broadly recognized as a general psychological vulnerability factor, associated with a heightened risk of depression, anxiety, and body-related concerns^19,20^. While not specific to body image disturbance, its association with emotional reactivity and interpersonal sensitivity may indirectly contribute to body dissatisfaction. Individuals high in neuroticism may be more likely to internalize appearance ideals and to engage in upward social comparisons, which can increase the perceived discrepancy between their actual and ideal physique^19,21^.

Extraversion, by contrast, appears to play a protective role^22^. Although associations between agreeableness, conscientiousness, and openness to experience with body image have been theorized, empirical findings are limited and inconclusive^17,22,23^. Some evidence suggests that agreeableness, through its link with interpersonal sensitivity and conformity to social norms, may increase vulnerability to body dissatisfaction when appearance-related norms are emphasized^22^. Openness may buffer against body image concerns through greater acceptance of diverse ideals^22^. Conscientiousness could have a dual role – encouraging health-promoting behaviours but might also be linked to greater sensitivity to appearance-related expectations^22,23^. However, most of these findings come from non-clinical populations.

Despite growing interest in the links between personality and body image, research has largely ignored the ICD-11 dimensional model of maladaptive personality traits. This model, now widely adopted in clinical diagnostics, captures extreme variants of FFM traits, offering a more nuanced and clinically relevant assessment framework. For example, negative affectivity mirrors high neuroticism, dissociality corresponds to low agreeableness, and anankastia reflects a rigid form of conscientiousness^24,25^.

Although previous research has linked ICD-11 maladaptive personality traits to eating pathology^26^, studies directly examining their associations with body image are lacking, particularly among clinical populations. This omission is striking given the increasing use of the ICD-11 model in psychological assessment. To our knowledge, the present study is the first to examine these relationships in the context of orthopedic surgery patients. Addressing this gap is essential for developing a comprehensive understanding of personality-based risk and protective factors related to body image.

### Orthopedic surgery and postoperative body image

Orthopedic surgery, especially in cases of trauma or joint replacement, causes substantial disruption to the body through surgical incisions, swelling, and restricted mobility. These procedures often result in visible scarring, asymmetries, or functional limitations, which can affect patients’ perception of their bodies and lead to dissatisfaction. In contrast, some interventions such as limb lengthening may enhance body image and self-esteem^27^. However, procedures involving physical changes, pain, and loss of autonomy may trigger body-related concerns and dissatisfaction, emotional distress, and a sense of disconnection from one’s body^28,29^.

Although body image has been examined in plastic, bariatric, and amputation-related surgeries^30–32^, orthopedic procedures remain underexplored in this context. Given their physical invasiveness and functional consequences, orthopedic surgeries may substantially impact patients’ body image and psychological well-being. Orthopedic surgery can pose a considerable risk for psychological distress, and in a notable proportion of patients may precipitate a mental health crisis. This risk is reflected in findings showing that psychological symptoms, such as anxiety, depression, and somatization, are frequently observed in orthopedic patients, both during the immediate recovery period and in the longer-term postoperative course^33,34^.

The early postoperative period can be particularly challenging. Studies suggest that patients experience high levels of pain, loss of independence, and discomfort in the first 72 h after surgery, a time during which they begin adapting to bodily changes and disruptions to their daily functioning^33,35^. In this context, body image becomes a psychologically meaningful construct that may influence emotional recovery, self-esteem, social reintegration, and engagement in rehabilitation.

## Materials and methods

### Study aim and conceptual framework

By focusing on a clinically relevant yet under-researched patient group and applying the ICD-11 dimensional model of personality pathology, this pilot study addresses a critical gap in the literature and aims to advance understanding of body image challenges and psychological risk factors in the early postoperative period following orthopedic surgery.

**Fig. 1:**
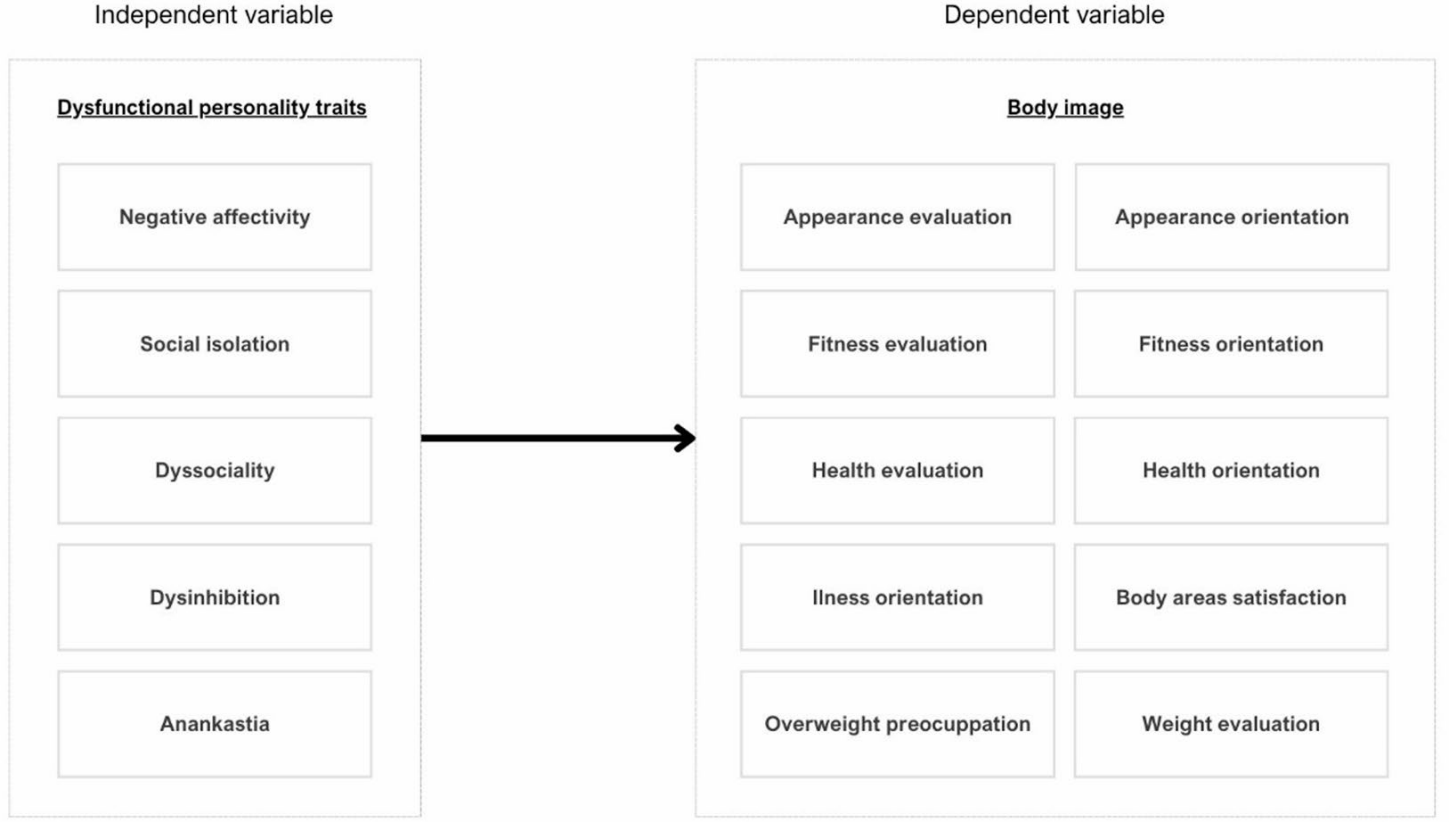
Research model.

To guide this investigation, a theoretical model was developed (Fig. 1), which illustrates hypothesized associations between maladaptive personality traits and body image dimensions in the early postoperative phase.

As shown in Fig. 1, five maladaptive personality traits - negative affectivity, disinhibition, anankastia, social isolation, and dissociality - are proposed to influence various cognitive, emotional, and behavioural aspects of body image. These include appearance and fitness evaluations, orientation toward health and fitness, satisfaction with specific body areas, and concerns about weight.

The independent variable in this study is the dysfunctional personality traits. It is understood as a set of traits describing excessive (maladaptive to maintaining health) intensification of basic personality traits. The variable consists of the following components:

- negative affectivity (a tendency to experience a wide range of negative emotions, the intensity and frequency of which are excessive for a given situation)^24,25^;
- disinhibition (a tendency to behave impulsively under the influence of immediate stimuli, without considering potential negative consequences)^24,25^;
- anankastia (a tendency to focus on rigid standards of perfection and a strong need to control one’s own and others’ behavior in order to conform to strict patterns)^24,25^;
- social isolation (the tendency to maintain distance in both interpersonal and emotional relationships; manifested by social withdrawal, and indifference to others)^24,25^;
- dissociality (the tendency to disregard social conventions, the rights and feelings of others, manifested in the ruthless pursuit of personal goals)^24,25^.

The dependent variable is body image, defined as a multidimensional construct that includes subjective thoughts, beliefs, feelings, and behaviours related to the body and physical functioning of the individual^1,2^.

The variable consists of the following components:

- appearance evaluation - level of subjective satisfaction/dissatisfaction with the body and perceived physical attractiveness/unattractiveness^1,36^;
- appearance orientation - level of focus on appearance and effort put into appearance;
- fitness evaluation - level of feeling physically fit^1,36^;
- fitness orientation - level of focus on physical fitness and effort put into being fit^1,36^;
- health evaluation - level of feeling physically healthy, being free of physical illness^1,36^;
- health orientation - the level of interest in, motivation for, and engagement with behaviors that promote physical well-being and a healthy lifestyle^1,36^;
- illness orientation - the degree of cognitive focus and emotional concern regarding one’s health status, including attentiveness to symptoms, vigilance toward bodily sensations, and readiness to detect or respond to signs of illness^1,36^;
- body area satisfaction - the level of satisfaction with particular body areas^1,36^;
- overweight preoccupation - the level of fear of gaining weight and the tendency to exhibit restrictive diet-related behaviors^1,36^;
- self-assessment of weight - the level of satisfaction with one’s own weight^1,36^.

The study’s primary research question is whether dysfunctional personality traits predict individual differences in body image following orthopedic surgery. Given the exploratory nature of this research, no formal hypotheses were proposed. However, based on prior findings in FFM literature, it was expected that negative affectivity would be linked to negative evaluations, and anankastia to increased orientation toward health and appearance-related behaviours^21,22^.

Understanding these relationships is important for identifying patients at risk for poor psychological outcomes. Insights into personality-based risk and resilience factors may inform psychoeducation and the development of tailored interventions that support patients’ adaptation and emotional recovery following orthopedic procedures.

### Participants and procedure

The study included 53 patients who had undergone orthopedic surgery (two patients were excluded from the analyses due to incomplete data). The surgeries the patients underwent were fracture fixation operations, joint endoprostheses, corrective osteotomies, and arthroscopic surgeries. While the type of orthopedic surgery was not formally controlled, all participants underwent musculoskeletal procedures involving the upper or lower limbs, requiring anesthesia, hospitalization, and postoperative rehabilitation. Participants were recruited using a convenience sampling method. Eligible patients were identified on the orthopedic ward and approached in person by trained members of the research team within 72 h post-surgery. Most participants underwent elective surgery (59%) under regional anesthesia (83%), and the majority (72%) received opioid analgesics postoperatively.

The subjects were selected based on specific inclusion criteria, including:

- age over 18 years-old;
- orthopedic surgery performed within 72 h prior to the participation in the study.

In addition, exclusion criteria were applied, which included:

- the presence or history of psychiatric disorders, such as anxiety, mood, eating, psychotic, personality or substance abuse disorders;
- cognitive impairments or postoperative disorientation that could interfere with participation; - limited proficiency in Polish or communication difficulties that would prevent understanding the study materials.

The study took place from May to November 2022 in the Department of Orthopedic and Trauma Surgery of the Zeromski Specialist Hospital in Krakow. The study was conducted in person by members of the research team and MSc students in psychology (under supervision, having received appropriate training in the research procedure to ensure consistent and reliable data collection) at the patients’ bedsides in the orthopedic ward. Each respondent was informed of the voluntariness of participation and the possibility of withdrawing from the study and provided written consent to participate. Before beginning the assessment, participants received verbal instructions and clarification of the procedure. The questionnaires (a metric, PiCD, and MBSRQ) were self-administered and typically required between 45 and 70 min to complete, depending on the patient’s condition. To accommodate postoperative fatigue or pain, patients were allowed to take breaks, complete the materials at their own pace, or receive support from the research team, including clarification of instructions. Researchers obtained permission from the Ethics Committee of the Jagiellonian University Institute of Psychology to conduct the study (decision number KE/3_2023). All methods were performed in accordance with the relevant guidelines and regulations.

### Materials

The Personality Inventory for ICD-11 (PiCD) **–** was created to measure pathological trait domains of personality according to the ICD-11 model. The PiCD consists of 60 items rated on a 5-point Likert scale (1 = “strongly disagree,” 5 = “strongly agree”) and measures five domains of dysfunctional personality traits: negative affectivity (15 items), social isolation (12 items), dissociality (12 items), disinhibition (9 items), and anankastia (12 items). The authors of the original version are Oltmanns and Widiger^24^. In the study, the tool was used in the Polish adaptation by Cieciuch et al.^25^. Cronbach’s alpha values for each subscale in the present sample were as follows: negative affectivity (α = 0.872), disinhibition (α = 0.731), social isolation (α = 0.687), dissociality (α = 0.822), and anankastia (α = 0.627).

Multidimensional Body Self Relations Questionnaire (MBSRQ) – was created to measure body image. The version used includes 10 subscales, each composed of items rated on a 5-point Likert scale (1 = “definitely disagree,” 5 = “definitely agree” or appropriate variants). The number of items per subscale is as follows: appearance evaluation (7 items), appearance orientation (12 items), fitness evaluation (3 items), fitness orientation (13 items), health evaluation (5 items), health orientation (8 items), illness orientation (5 items), body areas satisfaction (9 items), overweight preoccupation (7 items), and weight evaluation (2 items). The author of the original version is Cash^1^. In the study, the tool was used in the Polish adaptation of Brytek-Matera and Rogoza^36^. Cronbach’s alpha values for each subscale in the present sample were as follows: appearance evaluation (α = 0.795), appearance orientation (α = 0.697), fitness evaluation (α = 0.686), fitness orientation (α = 0.839), health evaluation (α = 0.641), health orientation (α = 0.653), illness orientation (α = 0.524), body areas satisfaction (α = 0.871), overweight preoccupation (α = 0.739), and weight evaluation (α = 0.902).

Metrics collecting sociodemographic data - in order to collect information such as age, sex, education, occupation, marital status, and type of orthopedic surgery, the authors developed a short metric that was filled out by patients at the beginning of the study. In addition, data regarding postoperative medication, type of anesthesia, and surgical procedure were obtained directly from medical staff, with patients’ consent.

### Data analysis

All analyses were conducted using IBM SPSS Statistics (version 27). Descriptive statistics, including means (M) and standard deviations (SD), were computed for all study variables. Prior to regression, the data were screened to verify key statistical assumptions. The Kolmogorov–Smirnov test indicated that distributions did not significantly deviate from normality (*p* > 0.05), and scatterplots were inspected for potential outliers, which were minimal and did not distort the results. Intercorrelations among predictors were examined; correlation coefficients below 0.30 suggested no relevant multicollinearity.

Next, a bivariate correlation analysis was conducted to explore the relationships between dysfunctional personality traits and body image components; the results are presented in Supplementary Table S1.

To identify significant psychological predictors of body image components, separate stepwise linear regression analyses were conducted for each outcome variable. The forward selection method was used, with predictors entered into the model at *p* < 0.05 and removed at *p* > 0.10. These thresholds reflect standard empirical criteria for balancing model parsimony with explanatory power.

To examine the robustness of the observed relationships, additional multiple regression analyses were performed including key clinical and demographic covariates: sex (0 = male, 1 = female), type of surgery (0 = elective, 1 = emergency), type of anesthesia (0 = regional, 1 = general), and pain medication (0 = non-opioid, 1 = opioid). These categorical variables were dummy-coded where applicable. Personality traits and clinical covariates were entered simultaneously into the models. The significance level was set at *p* < 0.05. Full results of the covariate-adjusted analyses are presented in the Supplementary Table S2 and Supplementary Table S3.

## Results

The study sample consisted of 53 patients. Descriptive statistics for sociodemographic data and study variables are provided in Tables 1 and 2.

**Table 1:** Characteristics of selected sociodemographic variables in the study group.

Variable*	Value	Descriptive statistics
Sex Female	*n* (%)	25 (47.2)
Male	*n* (%)	28 (52.8)
Education Primary	*n* (%)	3 (5.7)
Vocational	*n* (%)	9 (17.0)
Secondary	*n* (%)	19 (35.9)
Higher	*n* (%)	21 (39.6)
Status Married	*n* (%)	29 (54.7)
Partnership	*n* (%)	4 (7.6)
Single	*n* (%)	13 (24.5)
Widowed	*n* (%)	3 (5.7)
Divorced	*n* (%)	3 (5.7)
Age	*M ± SD* (min-max)	44.3 ± 14.20 (18,0–72.0)

*Categorical variables are expressed as frequency (*n*) and percentage (%), while variable age (continuous variable) is expressed as mean (*M)*, standard deviation (*SD)*, minimum (min) and maximum (max).

**Table 2:** Descriptive statistics for studied variables.

Questionnaire	Variable*	Value	Descriptive statistics
PiCD	Negative affectivity	*M ± SD* (min-max)	33.8 ± 8.7 (15.0–53.0)
	Dysinhibition	*M ± SD* (min-max)	23.7 ± 5.9 (13.0–37.0)
	Social isolation	*M ± SD* (min-max)	26.8 ± 5.7 (16.0–41.0)
	Dissociality	*M ± SD* (min-max)	23.3 ± 6.8 (14.0–44.0)
	Anankastia	*M ± SD* (min-max)	39.8 ± 5.2 (30.0–53.0)
MBSRQ	Appearance evaluation	*M ± SD* (min-max)	23.9 ± 4.9 (12.0–33.0)
	Appearance orientation	*M ± SD* (min-max)	22.6 ± 3.50 (16.0–29.0)
	Fitness evaluation	*M ± SD* (min-max)	10.13 ± 2.80 (3.0–15.0)
	Fitness orientation	*M ± SD* (min-max)	42.6 ± 9.19 (17.0–60.0)
	Health evaluation	*M ± SD* (min-max)	20.53 ± 4.48 (11.0–25.0)
	Health orientation	*M ± SD* (min-max)	26.6 ± 4.76 (15.0–40.0)
	Illness orientation	*M ± SD* (min-max)	16.0 ± 3.28 (9.0–25.0)
	Body areas satisfaction	*M ± SD* (min-max)	30.04 ± 6.71 (9.0–43.0)
	Overweight preoccupation	*M ± SD* (min-max)	10,43 ± 3,80 (7.0–18.0)
	Weight evaluation	*M ± SD* (min-max)	6.98 ± 1.66 (4.0–10.0)

*Variables are expressed in terms of mean (M), standard deviation (SD), minimum value (min) and maximum value (max).

To examine the predictors of body image components, a progressive stepwise regression analysis was conducted (see Table 3). The results show that dissociality was positively associated with appearance evaluation and fitness evaluation, explaining approximately 13% and 11% of the variance, respectively - representing moderate associations.

Negative affectivity was negatively associated with health evaluation (adjusted R² = 0.08) and positively associated with weight evaluation (adjusted R² = 0.09), both reflecting small effect sizes.

Anankastia was positively associated with health orientation and illness orientation, accounting for 7% and 6% of the variance, respectively - also indicating small associations.

**Fig. 2:**
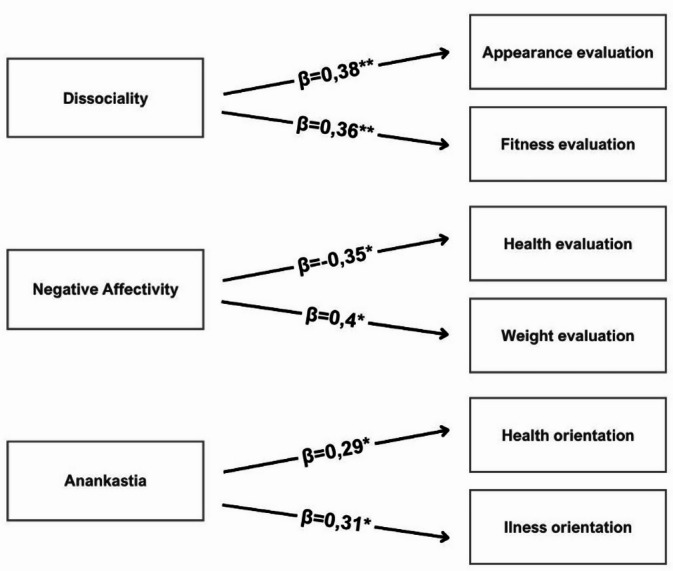
Model of research variables with graphical representation of β coefficients. Significance of β coefficients: *p < 0.05, **p < 0.01, ***p < 0.001.

Although the proportion of explained variance in each model ranges from small to moderate, all retained models met the threshold for statistical significance. The direction and relative strength of these relationships are illustrated in Fig. 2.

**Table 3:** Regression analysis for explained variables.

Explained variable	Predictors* (β, 95% CI)	Model summary
Appearance evaluation	Dissociality: 0.382**, 95% CI [0.088, 0.464]	Adjusted R² = 0.129, F(1,51) = 8.71, *p* < 0.01
Fitness evaluation	Dissociality: 0.358**, 95% CI [0.040, 0.257]	Adjusted R² = 0.110, F(1,51) = 7.50, *p* < 0.01
Health evaluation	Negative affectivity: − 0.346*, 95% CI [–0.325, − 0.31]	Adjusted R² = 0.077, F(2,50) = 3.17, *p* < 0.05
	Anankastia: 0.207, 95% CI [–0.067, 0.425]	
Health orientation	Anankastia: 0.289*, 95% CI [0.019, 0.512]	Adjusted R² = 0.066, F(1,51) = 4.67, *p* < 0.05
Illness orientation	Anankastia: 0.312*, 95% CI [0.023, 0.372]	Adjusted R² = 0.062, F(2,50) = 2.73, *p* < 0.05
	Social isolation: − 0.147, 95% CI [–0.244, 0.075]	
Weight evaluation	Negative affectivity: 0.404*, 95% CI [0.018, 0.136]	Adjusted R² = 0.090, F(2,50) = 3.58, *p* < 0.05
	Social isolation: − 0.265, 95% CI [-0.167, 0.013]	

Significance of β coefficients: *p < 0.05, **p < 0.01, ***p < 0.001.

CI: 95% confidence intervals for standardized β coefficients.

*The listed predictors were retained in the final stepwise regression models. Some variables did not reach statistical significance individually but were included based on the selection criteria used in the stepwise procedure.

Additional regression models were estimated controlling for clinical and demographic covariates. After covariates were entered, none of the personality traits remained statistically significant, although the directions of standardized β coefficients were consistent with the original models. Among the clinical predictors, pain medication reached significance (*p* = 0.041), and sex showed a trend-level effect (*p* = 0.054). Full results of the covariate-adjusted models are presented in the Supplementary Table S2 and Supplementary Table S3.

## Discussion

The present study indicates that certain dysfunctional personality traits are associated with specific components of body image. In particular, dissociality was positively related to both appearance evaluation and fitness evaluation. Individuals scoring high on dissociality, characterized by reduced concern for social norms and limited interpersonal sensitivity, may appraise their physical appearance and bodily fitness more favorably. This association might reflect a broader tendency to emphasize self-sufficiency and control, with physical attributes serving as indicators of personal autonomy, competence, or social value. While the exact mechanism remains unclear, previous research has shown that individuals with elevated dissocial traits, particularly those involving narcissistic features, tend to report higher self-perceived attractiveness and mate value, highlighting the potential role of appearance in self-presentation and social positioning^37^. However, it should be noted that the ICD-11 dissociality domain does not fully overlap with narcissism; rather, it encompasses a broader pattern of disregard for social conventions and others’ needs, which may only partially account for these associations.

Our study found that the trait of anankastia was positively associated with both health orientation and illness orientation. Individuals with elevated anankastic traits, characterized by a strong need for control and adherence to rigid personal standards, may report greater attentiveness to maintaining health and monitoring bodily symptoms. This tendency reflects a broader pattern of vigilance that extends into health-related behaviors. Previous research supports this notion, indicating that perfectionistic traits may have both adaptive and maladaptive implications for health. Harrison and Craddock^38^ observed that self-oriented perfectionism was associated with health-promoting behaviors, whereas socially prescribed perfectionism was linked to lower engagement. Further evidence suggests that perfectionistic tendencies may relate to pain perception and rehabilitation outcomes. In clinical samples, self-critical and socially prescribed perfectionism has been shown to exacerbate pain symptoms and impede functional recovery by increasing psychological stress and emotional distress during rehabilitation^39^. Although this finding originates from research on individuals with chronic pain, similar mechanisms may generalize to orthopedic surgery patients. In this context, anankastic traits may be related to how patients perceive pain, adhere to rehabilitation protocols, or evaluate treatment outcomes. This highlights the relevance of personality-based individual differences for postoperative care and underscores the need for further investigation in surgical populations.

Our results also indicated that negative affectivity was negatively associated with health evaluation and positively associated with weight evaluation. Individuals with a general tendency to experience a wide range of intense and persistent negative emotions tended to rate their overall health more negatively. This finding is consistent with previous research demonstrating that negative affect correlates with general dissatisfaction with life and various indicators of poor subjective health^40^.

An unexpected finding in this study was the positive association between negative affectivity and satisfaction with body weight. Typically, negative affect is associated with body dissatisfaction, particularly in domains such as weight evaluation^41^. However, some research suggests that individuals high in negative affect may engage in self-enhancement strategies that protect self-image, such as selectively focusing on areas they view more favorably or idealizing certain aspects of their appearance^42^. It is also possible that this pattern reflects an internalized need for control, where individuals experiencing heightened emotional distress may attempt to regulate their sense of order and self-efficacy through behaviors related to body weight and eating^43^. Although heightened emotional distress is not equivalent to the broader trait of negative affectivity, both involve sensitivity to negative emotional states and difficulties with emotion regulation, which may foster compensatory control-oriented behaviors. Such mechanisms could help explain why individuals with elevated negative affectivity report relatively higher satisfaction with their weight in the postoperative context.

This unexpected finding may also reflect characteristics specific to our clinical sample. In the context of orthopedic recovery, patients may view lower body weight as beneficial for regaining mobility or reducing joint strain^44^. As a result, individuals experiencing negative emotions might still report high satisfaction with their weight – not due to aesthetic approval, but because they perceive their current weight as helpful in the healing process. This interpretation reflects a functional, health-related perspective rather than an aesthetic one, emphasizing the clinical relevance of body image evaluations in postoperative care.

Factors such as gender distribution or cultural beliefs about health and body shape in orthopedic populations may also have influenced these results^45^. Further research is needed to clarify these associations, ideally using larger samples, longitudinal designs, and additional measures of coping styles, illness beliefs, and health-related values.

While the adjusted R² values in our models were generally low - ranging from approximately 6% to 13% - they reflect small to moderate effect sizes, which are common in psychological and clinical research, particularly in pilot studies with multifactorial constructs. Despite this, the associations observed remain theoretically meaningful and may carry clinical relevance: even weak statistical relationships can help identify patients at psychological risk and inform early supportive interventions. These results should therefore be interpreted with caution, but not disregarded, especially in light of their potential applicability in postoperative care planning.

The covariate-adjusted analyses confirmed that the observed relationships between personality traits and body image remained directionally consistent, although their statistical significance decreased once clinical factors were included. Among clinical variables, pain medication was a significant predictor, with patients receiving opioids reporting slightly higher appearance evaluation scores, possibly reflecting temporary improvements in comfort and body perception due to postoperative pain relief. A trend-level effect of sex suggested lower appearance satisfaction among women.

This reduction in significance is expected given the small sample size and the number of correlated predictors, which limited statistical power. Nevertheless, the stable effect directions support the robustness and theoretical coherence of the associations. These findings suggest that personality and clinical factors jointly influence postoperative body image, consistent with a biopsychosocial model. Future studies should verify these effects in larger samples with sufficient power to test potential mediating mechanisms.

### Limitations and strengths of the study, further research directions

The study presented here is not free of limitations. First, the study was conducted on a relatively small sample of patients. This reduces the possibility of generalizing the results, as well as the chance of detecting smaller but potentially meaningful associations between variables. However, it should be noted that the group of subjects in our study has its own specificity - it is a clinical sample, examined in a naturalistic hospital setting, and in a specific context (immediately after surgery). Access to patients in this postoperative phase is limited and challenging, as they often experience fatigue and pain, which may reduce willingness to participate in psychological research. Despite these constraints, we identified significant associations between dysfunctional personality traits and aspects of body image. This suggests promising directions for future research on psychological vulnerability in somatic contexts. The authors plan to conduct further studies with a larger sample of orthopedic patients to expand upon these preliminary findings.

Second, the limitations of the research are related to its cross-sectional nature. As the design is observational and correlational, no causal inferences can be drawn from the results. Although associations between personality traits and body image were identified, the direction of these relationships remains unclear. This design precludes conclusions about whether personality traits shape body image after surgery, or whether early postoperative experiences impact how traits are expressed or reported. A stronger emphasis must therefore be placed on cautious interpretation, as statistical associations do not imply temporal or causal direction. Moreover, the present study was conducted within 72 h after surgery, during the initial phase of recovery, when patients are typically still experiencing acute pain and beginning to adapt to changes in their body, functioning, and hospital environment^35,46^. While this timing is appropriate for capturing immediate psychological responses, we did not obtain preoperative data, nor did we include any follow-up measurement. As body image and emotional state likely evolve over time, we cannot determine whether the observed difficulties are transient or persistent. Ideally, future research would incorporate multiple, precisely timed assessments, e.g., preoperatively, and at 24, 48, and 72 h after surgery to more accurately capture postoperative dynamics.

Third, the study group included patients undergoing a variety of orthopedic procedures, including trauma-related surgeries (e.g., fracture fixation) and elective surgeries (e.g., arthroplasty, corrective osteotomies, arthroscopies). It is possible that patients with long-term pain and progressive loss of function experience their bodies differently than those who sustain a sudden injury. The psychological adjustment processes may also differ depending on the type and duration of the impairment. The authors are planning future studies to test these assumptions by examining more homogeneous subgroups.

It is also important to consider potential self-selection and social desirability biases. Since participation was voluntary and took place shortly after surgery, patients experiencing higher levels of distress may have declined to participate, limiting the representativeness of the sample. Additionally, the presence of researchers during questionnaire completion, despite the self-administered format, may have influenced patients’ responses toward socially acceptable answers.

Another methodological limitation involves the statistical approach. Although we used standard thresholds and validated instruments, some subscales demonstrated lower internal consistency values, which may have reduced measurement precision. In addition, due to the exploratory nature of the study and the relatively small sample size, no correction for multiple comparisons was applied. This increases the risk of Type I error and should be considered when interpreting the results. Finally, the use of stepwise regression, while useful for identifying potential predictors in pilot studies, has well-known limitations, including model instability and susceptibility to overfitting. Future studies with larger samples should consider using theoretically driven models and regularization techniques to improve generalizability.

Despite these limitations, the present study has several notable strengths. It is among the first to examine dysfunctional personality traits, operationalized via the ICD-11 dimensional model, in relation to body image in a surgical population. Prior studies exploring predictors of body image have primarily focused on normative personality traits or categorical personality disorders, whereas our approach offers a more dimensional and clinically applicable perspective. Furthermore, while the psychological consequences of orthopedic surgery have received some attention, studies specifically addressing body image in this patient group remain limited. Orthopedic patients are uniquely exposed to both visible and functionally disruptive bodily changes. Unlike other surgical specialties, orthopedic procedures often affect structural mobility and are directly linked to autonomy, movement, and daily functioning. Awareness of these limitations may affect self-esteem and satisfaction with one’s body, potentially contributing to emotional crisis and maladaptive adjustment.

The findings of this study highlight the psychological relevance of body image in orthopedic patients, particularly in the early postoperative period. Surgical procedures frequently involve visible and functional bodily changes that may provoke emotional discomfort, reduce self-esteem, and hinder recovery. These results underscore the need to address body-related concerns as part of comprehensive postoperative psychological care.

The findings of this study have potential clinical relevance in both health psychology and clinical psychology, particularly in informing early psychological screening and supportive interventions for orthopedic patients in the immediate postoperative period. Based on our findings, several clinical and preventive recommendations can be proposed. First, early personality screening, particularly for traits such as negative affectivity or anankastia, may help identify patients who are at greater psychological risk following surgery. Short screening tools derived from validated measures (e.g., PiCD) could be implemented in postoperative wards. Second, psychoeducational interventions delivered within the first 72 h after surgery may help normalize patients’ emotional reactions and body-related concerns, especially in individuals prone to perfectionism or emotional dysregulation. These could take the form of brief conversations, written materials, or supportive sessions conducted by nurses, physiotherapists, or psychologists. Third, patients with elevated maladaptive traits might benefit from early supportive interventions targeting emotional flexibility and acceptance of physical changes. Finally, for patients showing early signs of psychological distress, follow-up monitoring after discharge could help prevent longer-term difficulties in emotional adjustment or rehabilitation engagement.

It should be emphasized that these findings remain preliminary and should be interpreted with caution. Personality traits are not direct determinants of postoperative psychological outcomes, as these are shaped by multiple interacting variables, including pain levels, coping strategies, environmental support, and rehabilitation progress. Nonetheless, personality may function as a general psychological risk marker, helping to identify individuals more vulnerable to emotional distress or maladaptive body image responses. Rather than aiming to directly inform clinical interventions, the present study provides an exploratory framework for understanding how personality and body image interact in the acute post-surgical period. These findings may serve as a foundation for future hypothesis-driven research and the development of more refined psychological models in orthopedic care. To advance this line of research, future studies should prioritize larger and more homogeneous samples, employ longitudinal designs with repeated assessments (including preoperative baselines), and investigate potential mediators such as pain intensity, coping strategies, and perceived social support. Such efforts will help clarify the dynamic interplay between personality traits, body image, and recovery trajectories in orthopedic populations.

## Conclusions

Compared to previous literature focused mainly on non-clinical samples and normative personality traits, this pilot study offers a novel contribution by applying the ICD-11 model to examine body image in a clinical orthopedic population. There are significant associations between dysfunctional personality traits such as dissociality, anankastia, negative affectivity, and body image components among orthopedic surgery patients. Dysfunctional personality traits play a role in the perception and evaluation of one’s body image, but also in health/ illness orientation. The results of the present study may help in the development of preventive and therapeutic-support programs aimed at maintaining a healthy body image after orthopedic surgery.

## Supplementary Information

Below is the link to the electronic supplementary material.

Supplementary Material 1

## Data Availability

The dataset has been deposited in the RODBUK Cracow Open Research Data Repository and is available at the following link: https://doi.org/10.57903/UJ/H73AJO.
