# Supplementary material for: Dysfunctional personality traits and body image in orthopedic surgery patients at risk of postoperative psychological distress
Source: Sci Rep. 2025 Dec 24;15:44571. doi: 10.1038/s41598-025-28409-4 (PMC12739143; doi:10.1038/s41598-025-28409-4)
Supplement: Supplementary file 1 — Supplementary Material 1 [file 41598_2025_28409_MOESM1_ESM.docx]

*Supplementary Table S1.*

Pearson’s correlation coefficients between dysfunctional personality traits (PiCD subscales) and body image dimensions (MBSRQ subscales).

|  | Appearance Evaluation | Appearance Orientation | Fitness Evaluation | Fitness Orientation | Health Evaluation | Health Orientation | Illness Orientation | Body Areas Satisfaction | Overweight Preoccupation | Weight evaluation |
| --- | --- | --- | --- | --- | --- | --- | --- | --- | --- | --- |
| Negative Affectivity | –,054 | **,272*** | –,013 | –,036 | **–,273*** | ,123 | ,099 | ,021 | ,246 | ,270 |
| Disinhibition | –,002 | ,219 | ,089 | ,032 | –,252 | –,136 | –,137 | –,096 | ,160 | ,086 |
| Social isolation | ,044 | **,284*** | ,076 | ,101 | –,199 | ,060 | –,076 | –,001 | ,055 | –,059 |
| Dissociality | **,382**** | ,211 | **,358**** | ,220 | ,012 | –,052 | –,095 | ,180 | –,146 | –,102 |
| Anankastia | ,153 | ,173 | ,016 | ,090 | ,086 | **,289*** | **,279*** | ,159 | ,172 | ,102 |

Significance of Pearson’s r coefficients: *p<0.05, **p<0.01, ***p<0.001

*Supplementary Table S2.*

Clinical and demographic covariates predicting body image dimensions.

| **Dependent variable (MBSRQ subscale)** | **Sex β (p)** | **Type of surgery  β (p)** | **Type of anesthesia  β (p)** | **Pain medication  β (p)** | **Adj. R²** |
| --- | --- | --- | --- | --- | --- |
| **Appearance evaluation** | –0.343 (.054) | 0.140 (.320) | –0.092 (.523) | **0.284 (.041)*** | 0.205 |
| **Appearance orientation** | 0.137 (.483) | 0.226 (.155) | 0.101 (.532) | –0.065 (.668) | 0.000 |
| **Fitness evaluation** | –0.309 (.109) | –0.231 (.134) | 0.002 (.990) | 0.219 (.142) | 0.062 |
| **Health evaluation** | 0.004 (.982) | 0.288 (.064) | 0.095 (.543) | 0.105 (.477) | 0.060 |
| **Health orientation** | 0.151 (.457) | 0.206 (.210) | 0.110 (.510) | –0.137 (.385) | –0.072 |
| **Illness orientation** | –0.174 (.381) | –0.038 (.810) | 0.058 (.722) | –0.239 (.125) | –0.026 |
| **Body areas satisfaction** | –0.056 (.780) | 0.123 (.450) | –0.079 (.636) | 0.059 (.707) | –0.071 |
| **Overweight preoccupation** | 0.116 (.556) | 0.048 (.759) | 0.045 (.784) | –0.266 (.088) | –0.017 |
| **Weight evaluation** | 0.104 (.595) | 0.117 (.456) | 0.050 (.755) | –0.272 (.078) | 0.003 |

*Note.* Predictors entered simultaneously with personality traits (see Table 3).
p < .05*
Adjusted R² values correspond to each full model including both personality and clinical variables.

*Supplementary Table S3*

Personality traits (PiCD) predicting body-image dimensions after controlling for clinical covariates.

| **Dependent variable (MBSRQ subscale)** | **Neg. Affectivity β (p)** | **Disinhibition β (p)** | **Social Isolation β (p)** | **Dissociality β (p)** | **Anankastia β (p)** | **Adj. R²** |
| --- | --- | --- | --- | --- | --- | --- |
| **Appearance evaluation** | 0.148 (.531) | –0.082 (.668) | –0.137 (.427) | 0.318 (.062) | 0.199 (.193) | 0.205 |
| **Appearance orientation** | –0.013 (.960) | 0.140 (.517) | 0.223 (.253) | 0.138 (.463) | 0.046 (.785) | 0.000 |
| **Fitness evaluation** | 0.284 (.272) | –0.187 (.373) | –0.148 (.430) | 0.288 (.117) | 0.083 (.612) | 0.062 |
| **Health evaluation** | –0.308 (.234) | –0.020 (.925) | –0.028 (.881) | –0.009 (.960) | 0.223 (.181) | 0.060 |
| **Health orientation** | 0.024 (.930) | –0.087 (.696) | 0.154 (.443) | –0.027 (.890) | 0.120 (.497) | –0.072 |
| **Illness orientation** | 0.323 (.233) | –0.214 (.328) | –0.160 (.415) | –0.040 (.833) | 0.167 (.333) | –0.026 |
| **Body-area satisfaction** | 0.140 (.610) | –0.190 (.394) | –0.114 (.569) | 0.231 (.236) | 0.170 (.335) | –0.071 |
| **Overweight preoccupation** | 0.117 (.663) | 0.158 (.468) | 0.025 (.897) | –0.135 (.476) | 0.021 (.903) | –0.017 |
| **Weight evaluation** | 0.346 (.195) | –0.019 (.928) | –0.177 (.362) | 0.024 (.898) | –0.074 (.663) | 0.003 |

*Note.* Predictors entered simultaneously with clinical covariates (see Table 2).
None of the personality-trait effects reached statistical significance (p < .05).
Adjusted R² values correspond to each full model including both personality and clinical variables.
